# Effect of a Larger Flush Volume on Bioavailability and Efficacy of Umbilical Venous Epinephrine during Neonatal Resuscitation in Ovine Asphyxial Arrest

**DOI:** 10.3390/children8060464

**Published:** 2021-06-01

**Authors:** Deepika Sankaran, Payam Vali, Praveen Chandrasekharan, Peggy Chen, Sylvia F. Gugino, Carmon Koenigsknecht, Justin Helman, Jayasree Nair, Bobby Mathew, Munmun Rawat, Lori Nielsen, Amy L. Lesneski, Morgan E. Hardie, Ziad Alhassen, Houssam M. Joudi, Evan M. Giusto, Lida Zeinali, Heather K. Knych, Gary M. Weiner, Satyan Lakshminrusimha

**Affiliations:** 1 Department of Pediatrics, University of California, Davis, Sacramento, CA 95817, USA; pvali@ucdavis.edu (P.V.); pegchen@ucdavis.edu (P.C.); mehardie@ucdavis.edu (M.E.H.); zalhassen@ucdavis.edu (Z.A.); hmjoudi@ucdavis.edu (H.M.J.); egiusto@ucdavis.edu (E.M.G.); slakshmi@ucdavis.edu (S.L.); 2 Department of Pediatrics, University at Buffalo, Buffalo, NY 14203, USA; pkchandr@buffalo.edu (P.C.); sfgugino@buffalo.edu (S.F.G.); carmonko@buffalo.edu (C.K.); jhelman@buffalo.edu (J.H.); jnair@upa.chob.edu (J.N.); bmathew@upa.chob.edu (B.M.); mrawat@buffalo.edu (M.R.); lnielsen@buffalo.edu (L.N.); lizeinali@ucdavis.edu (L.Z.); 3 Department of Stem Cell Research, University of California, Davis, Sacramento, CA 95817, USA; allesneski@ucdavis.edu; 4 Department of Molecular Biosciences, Davis School of Veterinary Medicine, University of California, Davis, CA 95616, USA; hkknych@ucdavis.edu; 5 Department of Pediatrics, University of Michigan, Ann Arbor, MI 48109, USA; gweiner@med.umich.edu

**Keywords:** epinephrine, flush volume, neonatal resuscitation, chest compressions, asphyxia, cardiac arrest, epinephrine concentrations

## Abstract

The 7th edition of the *Textbook of Neonatal Resuscitation* recommends administration of epinephrine via an umbilical venous catheter (UVC) inserted 2–4 cm below the skin, followed by a 0.5-mL to 1-mL flush for severe bradycardia despite effective ventilation and chest compressions (CC). This volume of flush may not be adequate to push epinephrine to the right atrium in the absence of intrinsic cardiac activity during CC. The objective of our study was to evaluate the effect of 1-mL and 2.5-mL flush volumes after UVC epinephrine administration on the incidence and time to achieve return of spontaneous circulation (ROSC) in a near-term ovine model of perinatal asphyxia induced cardiac arrest. After 5 min of asystole, lambs were resuscitated per Neonatal Resuscitation Program (NRP) guidelines. During resuscitation, lambs received epinephrine through a UVC followed by 1-mL or 2.5-mL normal saline flush. Hemodynamics and plasma epinephrine concentrations were monitored. Three out of seven (43%) and 12/15 (80%) lambs achieved ROSC after the first dose of epinephrine with 1-mL and 2.5-mL flush respectively (*p* = 0.08). Median time to ROSC and cumulative epinephrine dose required were not different. Plasma epinephrine concentrations at 1 min after epinephrine administration were not different. From our pilot study, higher flush volume after first dose of epinephrine may be of benefit during neonatal resuscitation. More translational and clinical trials are needed.

## 1. Introduction

The International Liaison Committee on Resuscitation (ILCOR) advocates use of epinephrine in neonates with severe bradycardia (heart rate < 60 beats per minute [bpm]) despite effective positive pressure ventilation (PPV) and chest compressions (CC) if return of spontaneous circulation (ROSC) is not achieved [1,2]. Intravenous (IV) route is the preferred route for epinephrine administration due to greater efficacy and plasma epinephrine concentrations when compared to the endotracheal route [3,4,5]. In the delivery room, an umbilical venous catheter (UVC) can be inserted to 2–4 cm below the skin to allow quick administration of epinephrine. The 7th edition of the *Textbook of Neonatal Resuscitation* recommends 0.5-mL to 1-mL flush following IV epinephrine (0.01 to 0.03 mg/kg dose) via a low-lying UVC [6]. Although this flush volume may be sufficient in the setting of spontaneous cardiac activity (i.e., bradycardia), the recommended flush volume may only clear a 5 Fr UVC (internal volume = 0.55 mL) that is placed for term neonates and may not be sufficient to drive epinephrine to the heart and the circulating blood in the setting of cardiac arrest and CC [7]. Earlier ROSC following effective and quick delivery of an epinephrine dose by a route with maximum bioavailability may potentially improve survival and outcomes [8,9].

Recently, use of a 3-mL flush following IV or intraosseous (IO) epinephrine has been proposed by the American Academy of Pediatrics/American Heart Association (AAP/AHA) Neonatal Resuscitation Program (NRP) guidelines [10]. This recommendation is based on expert opinion and not based on robust scientific evidence. Our objective was to evaluate and compare the effect of different flush volumes of 1-mL and 2.5-mL following a 0.03 mg/kg epinephrine dose through a low UVC on the incidence of ROSC, and the incidence of ROSC after the first dose of epinephrine. We also evaluated the secondary outcomes of time to achieve successful ROSC and plasma epinephrine concentrations.

## 2. Materials and Methods

The current study protocol was approved by the Institutional Animal Care and Use Committee (IACUC) at the State University of New York, Buffalo, NY, USA (protocol PED10085N) and University of California Davis, Davis, CA, USA (protocol 20734). The experiments were performed in compliance with animal ethical guidelines (the ARRIVE guidelines) [11]. Time-dated healthy pregnant ewes from May Family Enterprises (Buffalo Mills, PA, USA) and Van Laningham Farms (Arbuckle, CA, USA) were fasted overnight and underwent cesarean section after endotracheal intubation under general anesthesia with IV diazepam and ketamine, and inhaled 2% isoflurane, as previously described [12,13].

### 2.1. Fetal Instrumentation

The fetal lamb was partially exteriorized for instrumentation while still attached to placental circulation. The lamb’s airway was intubated and the endotracheal tube was occluded. Carotid arterial and jugular venous catheters were inserted on the right sided blood vessels for preductal arterial blood draws, invasive blood pressure and heart rate monitoring, and IV access respectively. A flow probe (Transonics, Ithaca, New York, NY, USA) was placed around the left carotid artery to continuously measure blood flow.

### 2.2. Asphyxial Arrest and Resuscitation

The umbilical cord was compressed and occluded to induce asphyxia and cardiac arrest. Electrocardiogram leads (3- lead EKG) were applied. The lambs were resuscitated after 5 min of cardiac arrest (flat line on carotid arterial tracing and pulseless electrical activity of <20 bpm on EKG) per NRP guidelines. PPV was initiated with peak inflation pressures of 30–35 cm H_2_O, positive end expiratory pressure (PEEP) of 5 cm H_2_O and rate of 40 breaths per minute using 21% oxygen [14]. If the lambs did not achieve ROSC with effective PPV, then CC were initiated at 3:1 compression-to-ventilation ratio and supplemental oxygen was simultaneously increased to 100%. The lambs that did not have ROSC with PPV and CC alone and required IV epinephrine were included in the study. IV epinephrine (0.03 mg/kg/dose) was administered every 3 min via a low-lying UVC placed to a depth of 2–4 cm from the skin until ROSC was achieved. The epinephrine dose was followed by a flush volume of either 1-mL or 2.5-mL normal saline. The resuscitators were not blinded to the flush volumes. ROSC was defined as sustained spontaneous heart rate of >100 bpm along with a systolic blood pressure > 40 mm Hg. If ROSC was not achieved, cardiopulmonary resuscitation was continued for a total of 20 min with IV epinephrine repeated every 3 min for a maximum of 4 doses. Lambs were euthanized using IV pentobarbital (Fatal-Plus, Vortech Pharmaceuticals, Dearborn, MI, USA).

Arterial blood samples were obtained at the start of PPV and at 1 min after epinephrine and flush administration (since we anticipate peak plasma epinephrine concentrations at this time point). Plasma samples were frozen at −80 °C until analysis for epinephrine concentrations by ELISA (Eagle Biosciences, New York, NY, USA).

### 2.3. Primary and Secondary Outcomes

Primary outcome measures were incidence of ROSC and incidence of ROSC with the first dose of epinephrine.

Secondary outcome measures included time to achieve ROSC from time of epinephrine and flush, and plasma epinephrine concentration at 1 min after epinephrine and flush administration.

### 2.4. Data Collection and Statistical Analysis

Hemodynamic variables were continuously monitored during asphyxia, resuscitation and after ROSC, and recorded using BIOPAC systems (Goleta, CA, USA) software version- 4.3.1. Categorical data were analyzed by chi-squared test with Fisher’s exact test as required, non-parametric continuous variables by Mann–Whitney U test, and parametric continuous variables by unpaired *t*-test. Data were analyzed using Statview 5.0.1 (SAS Institute Inc., New York, NY, USA). Probability of <5% was used for statistical significance. Some of the data included in this manuscript were previously published [9,12].

Power calculation: Power was calculated for the parameter: incidence of ROSC with the first dose of epinephrine. We planned a study of 15 experimental subjects and 7 control subjects. Prior data indicate that the ROSC rate among controls is 0.40. If the true ROSC rate for experimental subjects is 0.80, we can reject the null hypothesis that the ROSC rates for experimental and control subjects are equal with probability (power) 0.47. The type I error probability associated with this test of this null hypothesis is 0.05.

## 3. Results

Twenty-two near-term lambs were asphyxiated until cardiac arrest by umbilical cord occlusion. Birth characteristics such as gestational age, birth weight, sex, and time to cardiac arrest from the time of umbilical cord occlusion were not different between the two study groups (Table 1).

### 3.1. Incidence of ROSC and Time to Achieve ROSC

Three out of seven (43%) and twelve out of fifteen (80%) lambs had ROSC after the first dose of epinephrine with 1-mL and 2.5-mL flush respectively (*p* = 0.08, Table 1). The time to achieve ROSC from the time of epinephrine administration was not different (*p* = 0.71, Table 1).

### 3.2. Cumulative Dose of Epinephrine and Epinephrine Concentrations in Plasma

The cumulative epinephrine dose required to achieve ROSC was not different with use of 1-mL and 2.5-mL flush volumes following the epinephrine dose (Table 1). Plasma epinephrine concentrations at 1 min after epinephrine and flush administration were also not different (Table 2).

### 3.3. Post-ROSC Hemodynamics

Heart rates, arterial blood pressures, and carotid blood flows at 10 min after ROSC were similar between the lambs that received 1-mL and 2.5-mL flush volumes after epinephrine via low UVC (Table 1).

## 4. Discussion

Perinatal asphyxia requiring extensive resuscitation including CC and epinephrine administration is associated with poor neurodevelopmental outcomes in neonates. Clinical measures to increase the incidence and hasten ROSC may potentially improve outcomes. The current study reports that larger flush volume of 2.5-mL normal saline following epinephrine at a dose of 0.03 mg/kg is associated with 80% incidence of ROSC, following the first dose of IV epinephrine, compared to 42% with the use of 1-mL flush.

**Figure 1:**
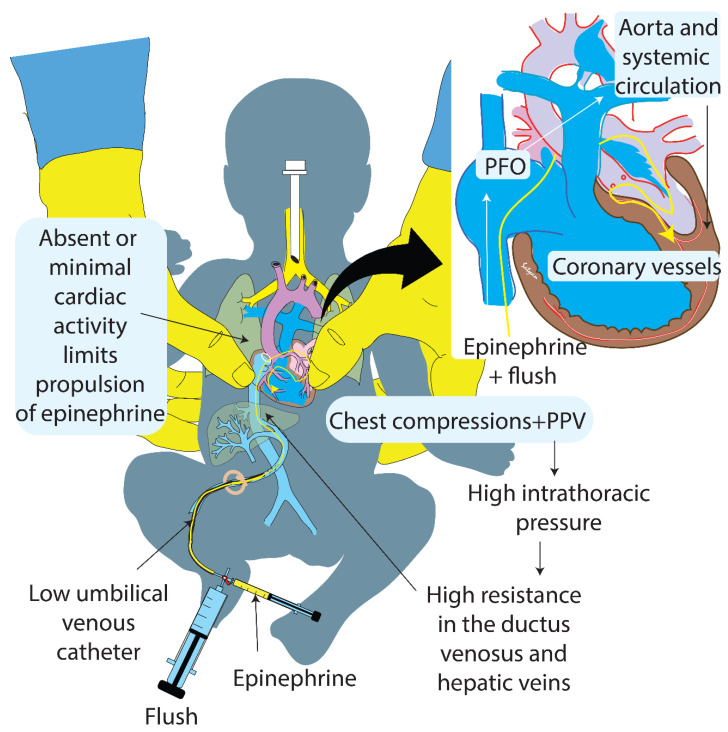
Schematic showing the speculative mechanism of larger flush volume following low umbilical venous catheter (UVC) epinephrine in term newborns. During cardiac arrest (absence of spontaneous cardiac activity) and during chest compressions and positive pressure ventilation (PPV) that increase the intrathoracic pressure and cause back pressure in the inferior vena cava, the epinephrine injected via a low umbilical venous catheter followed by a low flush volume of 0.5 mL to 1 mL may not be delivered to the right atrium. The epinephrine may be deposited in the umbilical vein or hepatic veins. The use of a higher flush volume may propel the epinephrine to the right atrium and across the patent foramen ovale (PFO) to left heart, aorta, systemic circulation, and coronary arteries increasing the chances of return of spontaneous circulation (ROSC). Inset shows a magnified view of the heart and coronary vessels. The yellow line represents the path of epinephrine and flush in the figure and the inset. Copyright Satyan Lakshminrusimha.

The 7th edition AAP–NRP *Textbook of Neonatal Resuscitation* recommended 0.5-mL to 1-mL of saline flush following epinephrine administration through a low umbilical venous route [6]. Due to lack of valves in the ductus venosus and high resistance in the ductus venosus during CC, epinephrine may be deposited in the umbilical vein and not reach the heart with 0.5 mL to 1 mL flush. Furthermore, epinephrine may increase the portal venous resistance, thus barricading the epinephrine within the portal venous system (Figure 1) [7]. A larger flush volume following low UVC epinephrine may maintain the patency of the ductus venosus and propel epinephrine into the right atrium. Contrast studies and angiography using a low UVC (inserted ~6 cm in a term newborn) in 1961 showed opacification of the left atrium and ventricle with contrast within seconds of injection [15]. We speculate that with adequate flush, epinephrine is propelled across the patent foramen ovale (PFO) to the left heart and systemic circulation (Figure 1). The AAP- NRP in the 8th edition of the Textbook of Neonatal Resuscitation have changed the recommendation and increased the flush volume to 3-mL following epinephrine [9,10].

We have previously compared the effect of flush volumes of 1-mL and ~10-mL after low UVC epinephrine in a term ovine model of perinatal cardiac arrest [12]. Larger flush volume of ~10-mL resulted in quicker ROSC when compared to 1-mL flush after the first epinephrine dose. However, use of flush volumes as high as ~10 mL may not be preferred by neonatal providers in the delivery room due to two reasons. Firstly, perinatal asphyxia can lead to myocardial dysfunction (systolic and diastolic) [16,17]. Excess volume administration during resuscitation may pose a strain on the already compromised heart, further increasing myocardial workload and oxygen demand [18,19]. Secondly, after prolonged asphyxia, hypercapnia and rapid increase in cerebral blood flow follows [20]. In this setting, further increases in cerebral blood flow due to excessive volume use during resuscitation may potentially worsen reperfusion injury. In extremely preterm infants, high flush volumes administered rapidly can lead to fluctuations in cerebral blood flow with a potential for severe intraventricular hemorrhage. In our previous experiment, we limited the larger flush volume to the first epinephrine dose. In contrast, in the current study, we used a 2.5-mL flush volume following subsequent doses of epinephrine as well.

Use of 1-mL flush had a trend towards lower incidence of ROSC following the first dose of epinephrine. Achieving early ROSC with the minimum required epinephrine doses may improve outcomes and avoid post-ROSC adverse effects of epinephrine including tachycardia, hypertension, and increased myocardial oxygen demand [21]. The current study did not demonstrate a difference in hemodynamic parameters of heart rate, arterial blood pressure, or carotid arterial blood flow at 10 min after ROSC when 1-mL and 2.5-mL flush volumes were used after the epinephrine dose. In addition to increasing efficacy of IV epinephrine, a larger flush volume may potentially decrease adverse effects by lowering the cumulative epinephrine dose required prior to achieving ROSC and warrants adequately powered clinical trials.

There are several limitations to this study. The included lambs were not randomized and the resuscitators were not blinded to the intervention. Furthermore, our study was underpowered to demonstrate a difference between the groups, and we may have seen a statistically significant difference if we had a larger sample size. We evaluated the effect of flush volume following low UVC epinephrine administration in a term large mammalian model of perinatal asphyxial arrest but did not evaluate preterm or bradycardia models. We have not evaluated the effect of flush volume following IO epinephrine. Species differences may result in different effects of flush volume in human neonates. Long-term cardiovascular and neurological outcomes were not evaluated. Real time physiological monitoring and epinephrine pharmacokinetics with plasma epinephrine concentrations are the strengths of this study. Furthermore, this is the first report evaluating the effects of using a 2.5-mL flush volume following a low UVC epinephrine in neonatal asphyxial arrest.

**Figure 2:**
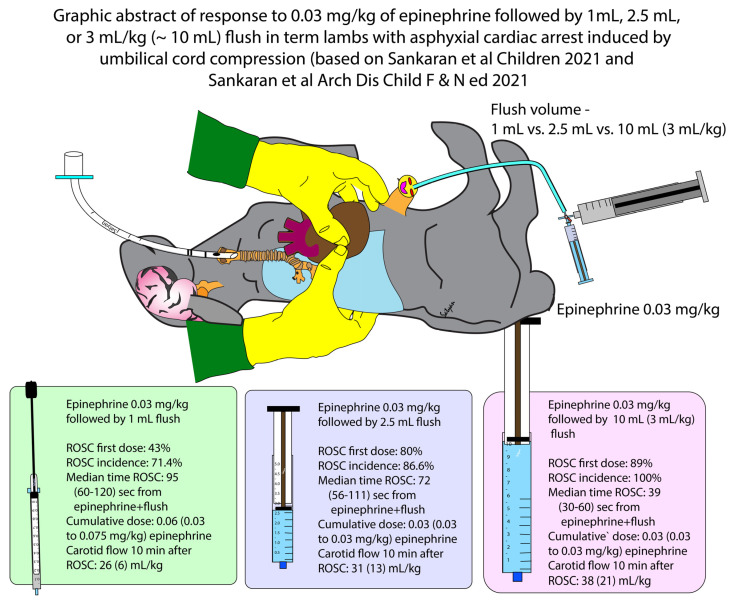
Graphic abstract of data from current study and Sankaran et al. [12] comparing 1-mL, 2.5-mL, and 3 mL/kg (~10 mL) flush in term lambs with asphyxial arrest induced by umbilical cord occlusion. In lambs receiving 10-mL flush, only the first dose of epinephrine was associated with high volume flush. (Copyright Satyan Lakshminrusimha).

Data from our previously published study evaluating 3 mL/kg flush volume (approximately 10-mL in term lambs) resulted in ROSC in 8/9 lambs with the first dose of epinephrine at 0.03 mg/kg (88%). In one lamb that did not achieve ROSC with the first dose, a subsequent dose of epinephrine 0.03 mg/kg with 1-mL flush led to ROSC. A graphic summary combining results from the current paper and our previous publication [12] of 3 doses of flush (1-mL, 2.5-mL, and 10-mL) is shown in Figure 2.

## 5. Conclusions

Larger flush volume following low UVC epinephrine may increase the incidence of ROSC with the first dose of epinephrine. Adequately powered clinical trials are warranted to study the effect of a larger flush volume following UVC epinephrine on survival and long-term cardiovascular and neurodevelopmental outcomes.

## Figures and Tables

**Table 1:** Comparison of characteristics between lambs that received 1-mL and 2.5-mL flush volumes after 0.03 mg/kg low UVC epinephrine.

Flush Volume	1-mL Flush *n* = 7	2.5-mL Flush *n* = 15	*p*-Value
Gestational age (days)	142 (2)	140 (1)	0.97
Weight (kg)	4.45 (1.3)	3.6 (0.8)	0.07
Sex distribution *n* (%)	4 females (57%)	6 females (40%)	0.45
Time to cardiac arrest (min)	14.7 (3.6)	15.6 (4.6)	0.89
ROSC incidence with the 1st dose of epinephrine *n* (%)	3 (42.8%)	12 (80%)	0.08
ROSC incidence *n* (%)	5 (71.4%)	13 (86.6%)	0.38
Median time to ROSC from time of epinephrine and flush (s)	95 (60–120)	72 (56–111)	0.71
Cumulative dose of epinephrine (mg/kg) median (interquartile range)	0.06 (0.03–0.075)	0.03 (0.03–0.03)	0.26
Mean blood pressure at 10 min after ROSC (mmHg)	64 (25)	65 (15)	0.26 0.96
Heart rate at 10 min after ROSC (beats per minute)	195 (14)	194 (13)	0.88
Left Carotid artery blood flow at 10 min after ROSC (ml/kg/min)	26 (6)	31 (13)	0.47

Data presented as mean (standard deviation) or median (interquartile range) as specified. Parameters were not different between the groups. Categorical data were analyzed by chi-squared test with Fisher’s exact test as required, non-parametric continuous variables by Mann–Whitney U test, and parametric continuous variables by unpaired *t*-test. UVC: umbilical venous catheter. ROSC: return of spontaneous circulation.

**Table 2:** Comparison of peak plasma epinephrine pharmacokinetics at 1 min following 1st dose of low UVC epinephrine at 0.03 mg/kg.

Parameter	1-mL Flush	2.5-mL Flush	*p*-Value
Plasma epinephrine concentration at 1 min after epinephrine dose among all the lambs studied (ng/mL).	494 (171)	519 (140)	0.92
Plasma epinephrine concentration at 1 min after epinephrine and flush among lambs that achieved ROSC with 1st dose (ng/mL)	572 (50)	545 (165)	0.94

Data are presented as mean (standard error of mean). Data not different by unpaired *t*-test.

## Data Availability

The data presented in this study are available in this article.
